# Integrating Problem-Based Learning Into an Internal Medicine Residency Curriculum

**DOI:** 10.31486/toj.22.0078

**Published:** 2022

**Authors:** Tiffany Wesley Ardoin, Diana Hamer, Michael Stumpf, Lauren Miles

**Affiliations:** ^1^ Department of Clinical Medicine, Louisiana State University Health Sciences Center, Baton Rouge, LA; ^2^ Department of Academic Research, Our Lady of the Lake Regional Medical Center, Baton Rouge, LA; ^3^ Department of Nephrology and Critical Care, Louisiana State University Health Sciences Center, Baton Rouge, LA

**Keywords:** *Education–medical*, *education–medical–graduate*, *problem-based learning*

## Abstract

**Background:** Problem-based learning (PBL) is a form of constructivist learning that allows learners to use higher order thinking by promoting learners to construct their own knowledge and understanding. PBL is prevalent in medical school education, but literature on PBL in graduate medical education (GME) is lacking. Because of the limited amount of data on PBL curricula in GME and the need for young physicians to develop critical thinking, lifelong self-directed learning, and problem-solving skills, we sought to incorporate PBL into the curriculum for our internal medicine residency program in a university-based community hospital setting.

**Methods:** The PBL committee created 4 cases derived from actual patient encounters that address common chief complaints encountered in the hospital and served as a crash course curriculum for interns in internal medicine. The success of the PBL curriculum was measured using a 39-question survey created by PBL leadership to assess the learners’ satisfaction with case content, likeability/design, feasibility, effectiveness, and motivation/self-learning. Additional questions asked for ways to improve PBL sessions in the future.

**Results:** Overall, interns felt the content was clinically relevant, challenged them to think critically, and aided in the medical management of their patients. They also found PBL to be more effective and more enjoyable than the traditional lecture-style curriculum.

**Conclusion:** Implementing a PBL curriculum in a residency program is possible. Although PBL has associated challenges such as scheduling, it is well received when supported by the program.

## INTRODUCTION

Problem-based learning (PBL), or case-based learning, has been a part of education in various health professions since the early 1990s.^1,2^ In addition to its use in allied health professional schools (ie, nursing, physical therapy, and occupational therapy), PBL has become increasingly more common in medical school education. PBL has been described as an effective educational style that promotes teamwork, self-directed learning, conceptual thinking, and interpersonal skills.^3,4^ Additionally, PBL has been shown to be effective in long-term knowledge retention, application of knowledge, and group learning.^5^ While PBL has been implemented in several medical schools throughout the United States and Europe to varying degrees, PBL is not common in graduate medical education (GME). Given the need for physicians-in-training to develop critical thinking, lifelong self-directed learning, and problem-solving skills, we sought to incorporate PBL into the curriculum for our internal medicine residency program in a university-based community hospital setting.

The structure of PBL varies from institution to institution.^6^ Most curricula reflect the design we used which involves separating learners into small groups led by a facilitator or tutor and sessions based on a clinical case or topic.^1^ Groups are given information related to a patient-based clinical case (ie, problem) in parcels to prompt discussion at significant points in the clinical scenario. The learners initiate and direct these discussions with the facilitator present to clarify information, referee discussion points, and move the discussion along if it stalls. The facilitators are not meant to be content experts or to lead the discussion. While each case has objectives, individual groups will have unique discussions. At the end of each session, each group develops individual topics for independent post-session learning in addition to the predetermined case objectives.

This case-based learning environment is the diametric opposite of traditional didactic teaching in which a content expert objectively discusses and transmits a topic with little to no input from learners. PBL is a form of constructivist learning that allows learners to use higher order thinking by promoting learners to construct their own knowledge and understanding. While both didactic teaching and PBL have their advantages, we felt that incorporating PBL in addition to the traditional lecture series would enhance the overall learning experience of the interns in an enjoyable way. Moreover, PBL uses many other adult learning theories through the focus on self-directed learning (humanistic learning theory); critical reflection on prior knowledge, leading to further acquisition and improvement of knowledge (transformative learning theory); and discussion among peers encouraging collaboration similar to future community practice (social theory of learning).^7^ Last, we anticipated that a PBL curriculum focused on common chief complaints would help interns enhance their knowledge and improve critical thinking early in their training. Although the PBL curriculum may positively impact interns’ self-directed learning and clinical skills, this report focuses on the feasibility and reception of the curriculum within the residency program.

## METHODS

### Case Development

The PBL leadership team developed 4 pilot cases representing common chief complaints encountered on medicine wards: chest pain, shortness of breath, abdominal pain, and encephalopathy. Portions of 2 cases are provided in Appendices 1 and 2. The cases, based on actual clinical cases from the previous year, were written by senior residents with details altered to remove patient identifiable information. While each case ended in a diagnosis related to the chief complaint, other objectives were included specific to each case. For example, the shortness of breath case included an arterial blood gas and challenged learners to develop an approach to acid-base derangements.

A content expert (general medicine faculty and subspecialists) reviewed each case to ensure that all content was correct and objectives were met. Cases consisted of chief complaint, history of present illness, past histories, physical examination, laboratory values, imaging, and other relevant diagnostic studies. At the end of each section of the case, learners were required to make a problem list and differential diagnosis, interpret laboratory and imaging studies, formulate a list of poorly understood topics for further reading, and identify the best next steps in diagnostics and management. Our intern groups often created concept maps to further illustrate their approach to the complaint and used Bayesian reasoning to deduce the final diagnosis from their list of differential diagnoses.

Each PBL case included a question regarding admission orders at a point relevant to each case. At this point, interns were encouraged to create admission orders for the patient in the case using an admission orders sheet created for the curriculum (Appendix 3). The intent of this unique addition to traditional PBL cases was to incorporate practical clinical skills that interns would use in addition to the educational approach to common diseases. Each step of the case also included questions derived specifically to help learners meet all learning objectives as they worked through the case.

Materials consisted of PBL case pages containing clinical details, a facilitator guide (consisting of the case information and suggested answers to clinical questions posed to help guide discussion), objectives, and teaching points for each case. The PBL case pages (without the facilitator guide answers) were provided to learners during the exercise. Facilitators had access to the entirety of the materials prior to and during the exercise. Case objectives and teaching points were provided to interns via email immediately following the exercise to encourage independent learning. The case objectives ensured that learners knew what material was intended to be covered. The teaching points included answers to clinical questions and concept maps illustrating the approach to the clinical topics, such as the approach to acute kidney injury or categories of diarrhea. During each session, supplemental materials, including electrocardiograms and relevant imaging such as chest x-rays, were available for learners to interpret.

### Session Design and Participants

The learners consisted of the 2018-2019, 2019-2020, and 2020-2021 intern classes (48 total interns) who were divided at random into 3 to 4 small groups of 3 to 5 people. A total of 4 sessions took place during the first 2 months of their intern year, occurring once every 1 to 2 weeks on Friday afternoons for 2 to 3 hours. Friday afternoons were chosen as this time had the least number of conflicts. During the first year, the sessions occurred weekly, but they were conducted every other week during the following years based on feedback from the learners. Small group member composition rotated based on call cycles and schedule conflicts, and facilitators rotated among groups to provide a diverse learning environment. Approximately 10 to 12 learners participated in each session based on call schedule. Interns were excused from the PBL session if it interfered with duty hours or clinical responsibilities. The PBL sessions occurred in small classrooms meant to accommodate no more than 12 learners at a time to ensure a comfortable, nonthreatening environment.

Facilitators consisted of selected senior residents with training in PBL, chief residents, and general medicine faculty on a volunteer basis. All facilitators received a brief training session from the PBL curriculum leader regarding the structure and nature of PBL, instructions on leading a PBL case, and specific case information. The training sessions included an opportunity to ask questions. Facilitators had access to the case information and objectives prior to each session.

At the beginning of the PBL session, each facilitator read a statement outlining the goals, process, and structure of the session (Appendix 4). During the first year, groups self-assigned 2 roles: quarterback and scribe. The role of the quarterback was to lead the group, read the session content, and guide the conversation. The scribe took notes on a whiteboard or windows with dry erase markers to keep track of the differential diagnoses, problem list, and further learning objectives. These roles were abandoned in later years based on feedback from the learners stating that they preferred multiple learners fill these roles during the sessions.

After the opening statement was read, the first page of the case was given to all learners to read and discuss. All small groups went through the same case at the same time. Relevant imaging, such as radiographic studies and electrocardiograms, was displayed on a computer monitor using PowerPoint (Microsoft Corporation) and in the individual case pages. After the facilitator assessed that all clinical questions had been thoroughly answered, the next page of the case was distributed. The only outside resources allowed were the American College of Physicians Medical Knowledge Self-Assessment Program 18 (ACP MKSAP 18) reference ranges for laboratory values (Appendix 5)^8^ and standard medical equations. Interns were encouraged to take notes during the case on other questions requiring research to allow for self-learning following the session. Additionally, a list of learning objectives and teaching points was emailed to all interns, including those unable to attend the session, following completion of the case to encourage further independent learning and to equip learners with solid learning resources covering common diagnoses they would encounter.

After the fourth PBL session, the interns were surveyed using an anonymous PBL Assessment Survey (Appendix 6). The PBL leadership team created this 39-question survey to assess the learners’ satisfaction with PBL case content, likeability/design, feasibility, effectiveness, and motivation/self-learning and to ask for ways to improve PBL sessions in the future. The survey was validated during the implementation of the first 15 surveys obtained in year 1 without the need for changes. Multiple authors initially reviewed the survey to determine content validity, and the survey proved to have adequate internal consistency (Cronbach α=0.68 with varying groups of questions). The survey was administered to all learners following completion of the PBL series. This study was granted exempt oversight by the Louisiana State University Health Sciences Center Institutional Review Board (IRB 538).

### Outcomes

The primary outcomes were based on learner opinion that the PBL content was clinically relevant, challenged them to think critically, and aided in the management of patients. Secondary outcomes included likeability, feasibility, and effectiveness of the PBL sessions. Secondary outcomes also included PBL sessions motivating learners to pursue self-learning.

### Statistical Analysis

SPSS Statistics, version 27 (IBM Corporation) was used for statistical analysis. For interpretation of the survey, we used descriptive statistics based on Likert scale and yes/no question mean values. These data were collected each July/August and analyzed during the following few months of that year.

## RESULTS

Among 48 interns, all interns attended at least 1 session. Thirty-seven were categorical and 11 were preliminary interns, with 35 males and 13 females. Forty (83.3%) participants completed the survey.

Regarding the assessment of the content of the PBL sessions, learners were overall pleased with the content. They felt that it was clinically relevant, challenged them to think critically, and aided in the medical management of patients with those chief complaints (Table 1). The mean scores were reflected in several learners’ answers to the free answer question following the first section: “For the PBL session that you found most beneficial, please describe why this session was the most beneficial.” Representative responses follow.

**Table 1.** Problem-Based Learning (PBL) Clinical Case Content Assessment, n=40

	Clinical Case			
Survey Question	Chest Pain	Shortness of Breath	Abdominal Pain	Encephalopathy
This PBL was clinically relevant.	4.74 (0.86)	4.79 (0.83)	4.73 (0.83)	4.96 (0.21)
This PBL challenged me to think critically.	4.70 (0.88)	4.67 (0.87)	4.77 (0.82)	4.83 (0.39)
This PBL aided in learning medical management of patients.	4.70 (0.88)	4.63 (0.88)	4.73 (0.83)	4.83 (0.39)
I found the teaching points and objectives helpful in answering clinical questions.	4.61 (0.89)	4.65 (0.88)	4.56 (0.92)	4.78 (0.42)

Notes: Response options were the following: 1=strongly disagree; 2=disagree; 3=neutral; 4=agree; 5=strongly agree. Data are presented as mean (SD).

*It provided me with a diagnostic framework in an area where I previously had no well-organized approach.*

*Clinically relevant to late night on-call situations.*

Further, learners who referred to the teaching points provided with the case found them useful in answering clinical questions.

Table 2 presents results from selected survey questions regarding likeability, feasibility, and effectiveness of the PBL curriculum. Overall, learners enjoyed PBL sessions; they did not prefer to use their time doing other residency-related work during the protected time for PBL sessions; and learners felt that PBL sessions were a more effective form of teaching than lectures. Although learners overall indicated that PBL sessions were a more effective learning modality compared to morning report, hospital rounds, and “chalk talks,” the agreements with these statements were only slightly above the response corresponding to “the same.” However, in the free text questions for the feasibility, effectiveness, and other sections of the survey, learners were enthusiastic about their PBL experiences. Representative responses follow.

**Table 2.** Assessment of Problem-Based Learning (PBL) Clinical Case Likeability, Feasibility, and Effectiveness, n=40

Assessment Category/Survey Question	Mean (SD)
Likeability	
I enjoyed the PBL sessions.^a^	4.69 (0.52)
Feasibility	
I would have rathered use my time for some other residency-related work or activity during this time.^a^	2.49 (1.15)
Effectiveness	
Since the PBL sessions, how often have you thought about the material discussed during PBLs when treating patients on wards?^b^	3.51 (0.70)
In your experience, how effective is PBL learning compared to lectures?^c^	4.36 (0.59)
In your experience, how effective is PBL learning compared to morning report?^c^	3.61 (0.80)
In your experience, how effective is PBL learning compared to hospital rounds?^c^	3.47 (0.84)
In your experience, how effective is PBL learning compared to brief lectures aka “chalk talks”?^c^	3.49 (0.74)

^a^Response options were the following: 1=strongly disagree; 2=disagree; 3=neutral; 4=agree; 5=strongly agree.

^b^Response options were the following: 1=never; 2=rarely; 3=sometimes; 4=often; 5=all of the time.

^c^Response options were the following: 1=much less effective; 2=less effective; 3=the same; 4=more effective; 5=much more effective.

*I think it's very helpful to be able to think out loud and be able to make mistakes.*

*These are MUCH more effective than lectures.*

*I like the PBLs a lot. There's nothing else I could’ve done from 1-3:30 every other Friday where I would have learned more.*

In years 2 and 3, we added questions to the survey to assess improved motivation and promotion of self-learning with PBL sessions. Therefore, only 25 residents answered these questions. Overall, 60% of the interns reported that they researched questions that came up during the PBL sessions. Moreover, after participating in PBL sessions, 60% of interns said they read about a topic they would not “have read about during intern year without exposure to PBLs.”

## DISCUSSION

This report demonstrates that a PBL curriculum can be integrated into GME and be well received. Although PBL curricula are common in undergraduate medical education, PBL is still rarely used at the graduate level, and limited data have been published on PBL curricula use in residency. Previous descriptions of PBL in residency report having the curriculum during one specific rotation of an internal medicine residency program.^9^ A 2001 description of a PBL curriculum integrated in a pediatric residency reported that the curriculum enhanced self-directed learning among participants in comparison to residents who only received traditional lectures.^3^ However, neither of these publications provided information on feasibility and likeability, nor did they provide guidance on how to replicate a PBL curriculum in other programs.

Through our implementation of PBL with our cohort of internal medicine interns, we found that the curriculum was well received. Learners felt the content was clinically relevant and that the active learning promoted by the PBL sessions challenged them to think critically and aided them in medical decision-making. Furthermore, the program was well-liked and a preferred method of learning over traditional lectures.

In the future, when we have a larger number of interns who have experienced the PBL curriculum, we will assess United States Medical Licensing Examination Step 1, 2, and 3 scores for current and previous interns to incorporate quantitative data regarding increased knowledge. Using Step 1 as a baseline comparison, we can determine if significant improvement was seen between Step 2 and Step 3 scores in classes exposed to PBL vs those not exposed to PBL. Hoffman et al used this technique to assess the PBL curriculum at the University of Missouri in 2006.^10^

Implementing a PBL program into a GME setting was more challenging than in other settings previously described because of the significant time demands of clinical duties and previously implemented protected learning time such as morning reports, noon conferences, and simulation laboratories. However, carving out 2 to 3 hours for 4 Friday afternoons in July and August for interns was feasible for our program because of the unwavering support of our program leadership. Further, creating cases addressing common chief complaints on wards and incorporating admission orders into the case catered to the unique needs of internal medicine interns.

Limitations include that this study was small, observational, and limited to a single university-based internal medicine program. Programs with more residents may be able to incorporate this curriculum more easily. Also, programs with different schedules may face different challenges. More research needs to be done on the feasibility and effectiveness of incorporating PBL and other forms of active learning within GME.

The current curriculum includes 4 cases designed for beginning interns, but we have started to expand the curriculum to include more complex cases for second- and third-year residents. We will then survey the residents to determine if they still think PBL is valuable to their learning. PBL may prove to be more impactful for learners early in their training who are looking to acquire clinical approaches to common problems. However, PBL may be just as beneficial or even more beneficial as learners gain more critical thinking skills as they progress through residency and look for more advanced exercises to practice these skills in a controlled environment. As with all educational endeavors within GME, there is a balance between service and education, and we must be mindful to allow learners to provide feedback on their learning experiences so that balance is not disrupted.

## CONCLUSION

This observational study suggests that implementing a PBL curriculum in an internal medicine residency program intern year was enjoyable and possibly more effective than traditional lectures. Interns felt that the content, which was based on common internal medicine ward chief complaints, was clinically relevant, challenged them to think critically, and aided in the medical management of their patients. We hope this publication can assist other GME programs in adopting a PBL curriculum.

## Notes

*This article meets the Accreditation Council for Graduate Medical Education and the American Board of Medical Specialties Maintenance of Certification competencies for Patient Care, Medical Knowledge, and Practice-Based Learning and Improvement.*

## References

[1] Davis MH. AMEE Medical Education Guide No. 15: problem-based learning: a practical guide. Med Teach. 1999;21(2):130-140. doi: 10.1080/01421599979743 21275726

[2] Strobel J, van Barneveld A. When is PBL more effective? A meta-synthesis of meta-analyses comparing PBL to conventional classrooms. Interdiscip J Probl-based Learn. 2009;3(1):44-58. doi: 10.7771/1541-5015.1046

[3] Ozuah PO, Curtis J, Stein RE. Impact of problem-based learning on residents' self-directed learning [published correction appears in Arch Pediatr Adolesc Med 2001 Dec;155(12):1350]. Arch Pediatr Adolesc Med. 2001;155(6):669-672. doi: 10.1001/archpedi.155.6.669 11386954

[4] Wood S. Views of the effectiveness of problem-based learning. Nurs Times. 2006;102(21):34-38. 16755808

[5] Yew EHJ, Goh K. Problem-based learning: an overview of its process and impact on learning. Health Prof Educ. 2016; 2(2):75-79. doi: 10.1016/j.hpe.2016.01.004

[6] Scholkmann A. Why don’t we all just do the same? Understanding variation in PBL implementation from the perspective of translation theory. Interdiscip J Probl-based Learn*.* 2020*;* 14 *(*2*). doi:* 10.14434/ijpbl.v14i2.28800

[7] Taylor DC, Hamdy H. Adult learning theories: implications for learning and teaching in medical education: AMEE Guide No. 83. Med Teach. 2013;35(11):e1561-e1572. doi: 10.3109/0142159X.2013.828153 24004029

[8] American College of Physicians. Reference Ranges MKSAP 18. annualmeeting.acponline.org/sites/default/files/shared/documents/for-meeting-attendees/reference-ranges-table.pdf

[9] Foley RP, Poison AL, Vance JM. Review of the literature on PBL in the clinical setting. Teach Learn Med. 1997;9(1):4-9. doi: 10.1080/10401339709539805

[10] Hoffman K, Hosokawa M, Blake R Jr, Headrick L, Johnson G. Problem-based learning outcomes: ten years of experience at the University of Missouri-Columbia School of Medicine. Acad Med. 2006;81(7):617-625. doi: 10.1097/01.ACM.0000232411.97399.c6 16799282

